# Assessing the impact of antiretroviral therapy on tuberculosis notification rates among people with HIV: a descriptive analysis of 23 countries in sub-Saharan Africa, 2010–2015

**DOI:** 10.1186/s12879-018-3387-z

**Published:** 2018-09-26

**Authors:** Diya Surie, Martien W. Borgdorff, Kevin P. Cain, Eleanor S. Click, Kevin M. DeCock, Courtney M. Yuen

**Affiliations:** 1 Division of Global HIV and TB, Centers for Disease Control and Prevention, Atlanta, GA USA; 2 Center for Global Health, Office of the Director, Centers for Disease Control and Prevention, Kisumu, Kenya; 3 Division of Global HIV and TB, Centers for Disease Control and Prevention, Kisumu, Kenya; 4 Division of Global HIV and TB, Centers for Disease Control and Prevention, Nairobi, Kenya; 5 Division of Global Health Equity, Brigham and Women’s Hospital, Boston, MA USA; 6 Department of Global Health and Social Medicine, Harvard Medical School, Boston, MA USA

**Keywords:** Tuberculosis, HIV infections, Incidence, Epidemics, Africa, Risk factors

## Abstract

**Background:**

HIV is a major driver of the tuberculosis epidemic in sub-Saharan Africa. The population-level impact of antiretroviral therapy (ART) scale-up on tuberculosis rates in this region has not been well studied. We conducted a descriptive analysis to examine evidence of population-level effect of ART on tuberculosis by comparing trends in estimated tuberculosis notification rates, by HIV status, for countries in sub-Saharan Africa.

**Methods:**

We estimated annual tuberculosis notification rates, stratified by HIV status during 2010–2015 using data from WHO, the Joint United Nations Programme on HIV/AIDS, and the United Nations Population Division. Countries were included in this analysis if they had ≥4 years of HIV prevalence estimates and ≥ 75% of tuberculosis patients with known HIV status. We compared tuberculosis notification rates among people living with HIV (PLHIV) and people without HIV via Wilcoxon rank sum test.

**Results:**

Among 23 included countries, the median annual average change in tuberculosis notification rates among PLHIV during 2010–2015 was -5.7% (IQR -6.9 to -1.7%), compared to a median change of -2.3% (IQR -4.2 to -0.1%) among people without HIV (*p*-value = 0.0099). Among 11 countries with higher ART coverage, the median annual average change in TB notification rates among PLHIV was -6.8% (IQR -7.6 to -5.7%) compared to a median change of -2.1% (IQR -6.0 to 0.7%) for PLHIV in 12 countries with lower ART coverage (*p* = 0.0106).

**Conclusion:**

Tuberculosis notification rates declined more among PLHIV than people without HIV, and have declined more in countries with higher ART coverage. These results are consistent with a population-level effect of ART on decreasing TB incidence among PLHIV. To further reduce TB incidence among PLHIV, additional scale-up of ART as well as greater use of isoniazid preventive therapy and active case-finding will be necessary.

## Background

Human immunodeficiency virus (HIV) infection is the most powerful known risk factor for tuberculosis (TB). HIV increases both the risk of reactivation of latent TB infection and the risk of rapid progression to active TB disease [1–4]. At the population level, the increased risk of TB among people living with HIV (PLHIV) has resulted in the resurgence of TB epidemics worldwide [5, 6]. Nowhere has this convergence of epidemics been more pronounced than in sub-Saharan Africa where HIV remains a major driver of the TB epidemic [7–9]. While only 10% of 10.4 million new TB cases reported worldwide in 2016 were among PLHIV, almost three quarters of these cases occurred in sub-Saharan Africa [9]. Successful TB control in this region requires addressing the disproportionate burden of TB among PLHIV.

In the last decade, the use of antiretroviral therapy (ART) in sub-Saharan Africa has greatly increased [10]. By helping to restore the immune system, ART has been shown to have a substantial effect on preventing TB in PLHIV [11–14]. In a meta-analysis of 11 studies, participants receiving ART had a 65% reduction in the development of TB compared to participants receiving no ART, regardless of their baseline CD4 count [11]. Given its proven individual-level effect, one would expect that the expansion of ART coverage would lead to the population-effect of declining TB incidence among PLHIV. Indeed, in Kenya, South Africa, and Malawi, TB notification rates among people with HIV are estimated to have declined substantially more than TB notification rates among people without HIV, concurrent with the expansion of ART coverage [15–18]. However, the extent to which this is true across the sub-Saharan African region is unclear.

To examine whether there is evidence of a population-level effect of ART on TB across sub-Saharan Africa, we estimated TB case notification rates stratified by HIV status for countries in the WHO African region. We then sought to compare trends in case notification rates among PLHIV and people without HIV, assessing both in the context of changing ART coverage.

## Methods

### Data sources

We estimated TB case notification rates stratified by HIV status using several existing, publicly available data sources. We obtained the number of total notified TB cases in each country, the number with HIV test results, and the number with positive HIV test results, by year, from WHO [19]. We obtained HIV prevalence estimates among adults aged 15–49 years, by year, from the Joint United Nations Programme on HIV/AIDS (UNAIDS) [20], and population estimates among adults aged 15–49 years, by year, from the United Nations Population Division (UNPD) [21].

To describe trends in ART coverage, we obtained ART coverage estimates, by year, from UNAIDS [22]. Coverage is defined as the percentage of all adults and children living with HIV who are currently receiving ART [23]. At the time of publication, ART coverage estimates from UNAIDS were available from 2010 onwards, and population estimates from UNDP were available through 2015; the period of our analysis was therefore from 2010 through 2015.

### Inclusion and exclusion criteria

All 47 countries in the WHO African region were considered for this analysis. We considered data quality for each country and each year to determine inclusion. A year of data was considered to be of adequate quality if an HIV prevalence estimate was available and if ≥75% of notified TB cases had been tested for HIV. Any country with <4 years of data meeting these criteria was excluded from analysis. After estimating HIV-stratified TB notification rates, we excluded countries that showed greater than 50% year-to-year variation in the estimated notification rates, suggesting either data quality issues or too few patients with TB to make rate calculations meaningful.

### Estimating TB notification rates stratified by HIV status

**Fig. 1:**
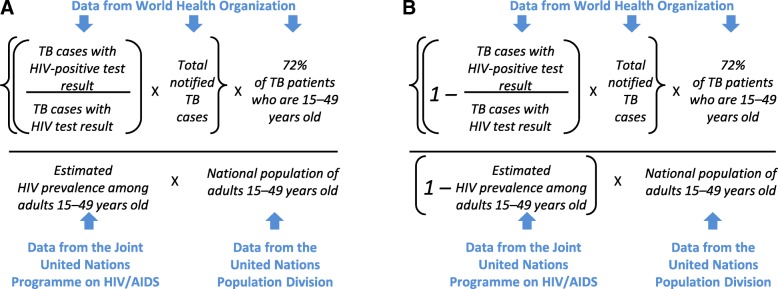
**a** and **b** Calculation of estimated annual TB notification rates for (**a**) people living with HIV and (**b**) people without HIV

To estimate TB notifications among PLHIV, we estimated a numerator based on TB notifications and a denominator based on population-level HIV-prevalence estimates (Fig. 1a). For the numerator, we estimated the annual number of notified TB cases in PLHIV in a country in a given year by multiplying the number of total notified TB cases in the country by the proportion of TB cases with an HIV-positive test result. We therefore assumed that TB cases who did not receive an HIV test had an equal likelihood of being HIV-positive as those who did; we believe this assumption to be reasonable for the countries included in our analysis given that routine HIV testing for TB patients has been recommended since 2004 [24] and that we only included countries where ≥75% of TB patients had been tested. We used aggregate TB case notifications instead of age-stratified TB case notifications due to inconsistencies in the age-stratified data. For the denominator, we calculated the number of PLHIV in each country by multiplying UNAIDS HIV prevalence estimates among adults aged 15–49 years by the UNPD population estimate for this age group. We restricted this population denominator to adults aged 15–49 years, as the HIV prevalence estimates are more robust for this age group than for children or older adults. To address the mismatch between the age groups in the numerator and denominator, we multiplied the numerator by the median average proportion of notified TB cases that were aged 15–49 years old among 19 countries in which the sum of all reported TB cases with a known age was ≥90% of all reported TB during 2010–2015 (median: 72%, interquartile range [IQR]: 69–75%).

A similar process was used to estimate TB notification rates among people without HIV (Fig. 1b). We subtracted the calculated TB/HIV cases from total notified TB cases to obtain the numerator, multiplying by 72% to correct for the mismatch in age groups between numerator and denominator. We subtracted the population of adults 15–49 years old with HIV from the total population in this age group to form the denominator.

### Trends in TB notification rates

To describe trends in TB notification rates during 2010–2015, we calculated for each country the average annual percent change in TB notification rate among PLHIV and the average annual percent change in TB notification rate among people without HIV. These calculations were based on the corresponding 2010 and 2015 notification rates and assumed a constant annual percent change during this period. That is:

$$ N=\mathrm{M}\times {\left(1+A\right)}^4 $$

Where:

N: = case notification rate in 2015
M: = case notification rate in 2010
A: = average annual percent change in case notification rate

For countries that lacked data for either 2010 or 2015, we performed an analogous calculation using notification rates for the terminal years of the date range.

Because of the potential error introduced by the assumptions we made in estimating TB case notification rates stratified by HIV status, we focused our analysis on assessment of trends over time, reasoning that even if the estimates for a country were inaccurate in a systematic way, the relationship between estimates in different years would remain robust. We used the Wilcoxon rank sum test with exact *p*-values to compare whether average annual changes in TB notification rates among PLHIV were significantly different from average annual changes in TB notification rates among people without HIV across all countries. To determine whether declines in TB notification rates among PLHIV were greater in countries with higher ART coverage, we compared the countries with greater-than-average ART coverage to those with lower-than-average ART coverage using the Wilcoxon rank sum test. The same comparison was made for declines in TB notification rates among people without HIV. Data were analyzed using SAS version 9.3 (SAS Institute, Cary, NC).

## Results

### Countries included in analysis

**Fig. 2:**
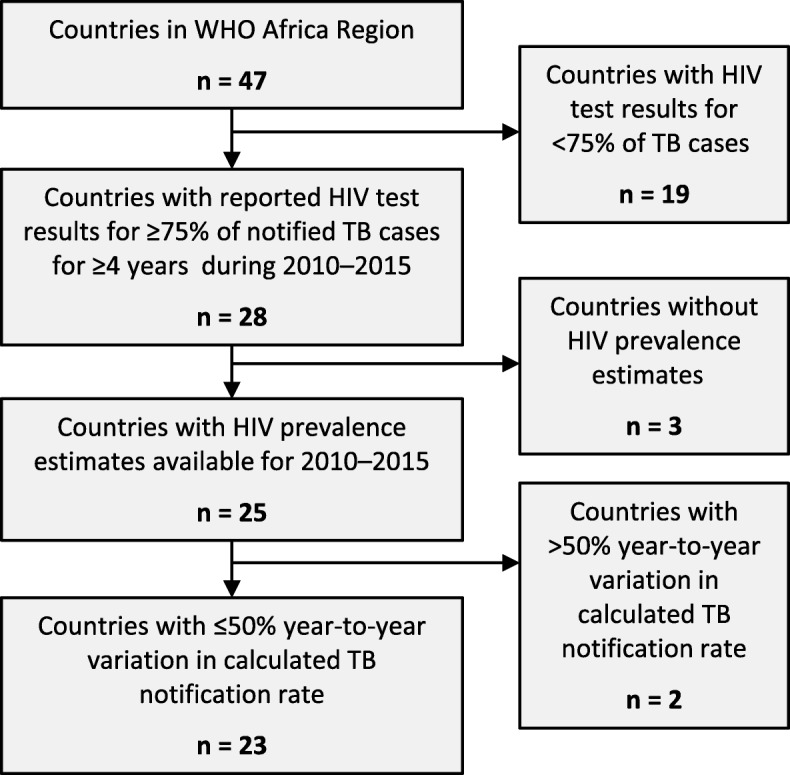
Flowchart depicting selection of countries for this analysis

**Fig. 3:**
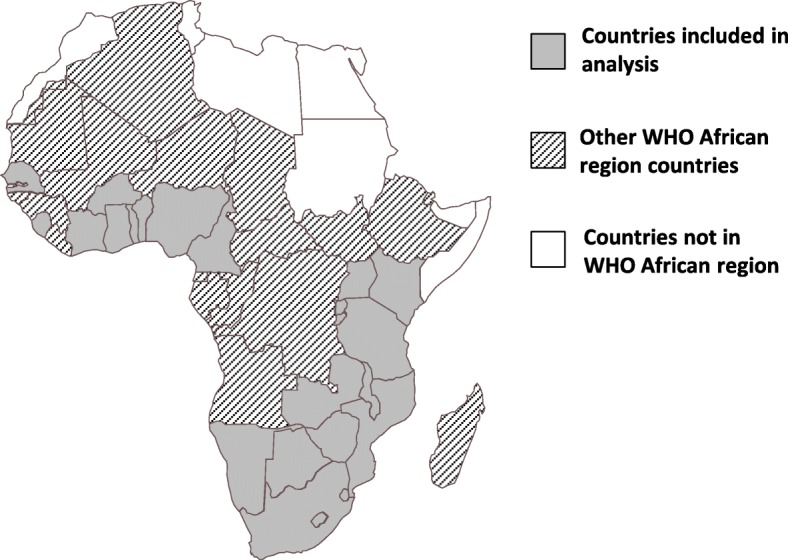
Countries included in analysis — sub-Saharan Africa, 2010–2015, (*n* = 23)

Of 47 countries in the WHO African region, 23 (49%) met inclusion criteria (Figs. 2 and 3) with 156 individual years of data included across the 23 included countries. In 2010, the median HIV prevalence in these countries was 5.6% (IQR 1.9–14.2%) and median WHO estimated TB incidence was 219 cases per 100,000 population (IQR 133–633 cases per 100,000 population; Table 1). In 2010, the median ART coverage was 26% (IQR 16–33%). From 2010 to 2015, the median absolute increase in ART coverage, defined as the difference between ART coverage in 2015 and ART coverage in 2010, was 25% (IQR 16–31%). Across all 156 years of included data, the median HIV testing coverage among TB cases was 96% (IQR 90–99%).

**Table 1:** TB and ART trends: comparative summary statistics — 23 countries, sub-Saharan Africa, 2010–2015

	2010 estimated adult HIV prevalence, % (95% CI)	2010 estimated TB incidence per 100,000 (95% CI)	2010 ART coverage,%	Absolute increase in ART coverage^b^, %	Average ART coverage, %	Average annual percent change in TB notification rate^a^	
						People with HIV (%)	People without HIV (%)
Central Africa							
Cameroon	4.6 (4.0–5.1)	274 (226–327)	16	14	23	−1.7	+ 1.2
Eastern Africa							
Burundi	1.5 (1.1–1.8)	144 (127–163)	25	25	36	−6.2	−0.9
Kenya	6.1 (5.5–6.8)	298 (286–311)	29	28	42	−6.8	−3.9
Rwanda	3.5 (2.9–4.0)	106 (94–118)	47	27	61	−5.7	−4.1
Tanzania	5.6 (4.7–6.4)	177 (166–189)	18	37	36	−1.4	−2.3
Uganda	7.4 (6.9–7.8)	209 (169–253)	20	40	39	−5.5	−0.1
Southern Africa							
Botswana	23.0 (20.3–24.4)	500 (449–560)	50	27	63	−6.8	−4.7
Lesotho	23.3 (21.1–24.5)	633 (553–719)	29	11	33	−8.3	−5.1
Malawi	10.9 (9.9–11.3)	219 (203–236)	26	32	43	−6.9	−4.2
Mozambique	14.0 (12.3–15.6)	544 (377–741)	13	31	25	+ 1.4	+ 9.3
Namibia	14.2 (12.5–15.5)	867 (686–1070)	43	20	55	−7.6	−0.1
South Africa	18.3 (15.9–20.5)	981 (809–1170)	20	29	36	−8.1	−1.3
Swaziland	27.2 (24.8–28.5)	1290 (1060–1530)	33	36	49	−10.9	−7.4
Zambia	12.7 (12.1–13.3)	462 (418–509)	34	30	49	−6.3	−3.7
Zimbabwe	15.8 (13.5–18.2)	633 (489–795)	29	39	49	−7.6	−5.0
Western Africa							
Benin	1.1 (0.8–1.5)	69 (57–82)	33	18	42	−0.5	−1.8
Burkina Faso	1.1 (0.8–1.3)	58 (49–67)	31	24	42	−6.3	+ 1.9
Côte d’Ivoire	3.8 (3.2–4.2)	190 (165–217)	16	18	24	−2.5	−2.8
Ghana	1.9 (1.5–2.4)	86 (75–97)	13	15	23	−2.0	−3.4
Nigeria	3.4 (2.4–4.6)	133 (64–225)	11	15	18	−5.2	−0.1
Senegal	0.6 (0.5–0.7)	137 (113–163)	28	16	34	−2.2	+ 2.7
Sierra Leone	1.7 (1.3–2.1)	660 (540–791)	11	10	15	+ 2.0	−4.2
Togo	2.9 (2.1–4.2)	73 (60–87)	19	20	28	+ 3.3	−2.2

^a^Average annual percent change is calculated by assuming a constant annual percent change between the first and last years of TB case notification rate estimates based on available high-quality data during 2010–2015; positive values indicate an increase in TB incidence while negative numbers indicate a decrease

^b^Absolute increase in ART coverage is defined as the difference between ART coverage in 2015 and ART coverage in 2010

### Trends in TB notifications

**Fig. 4:**
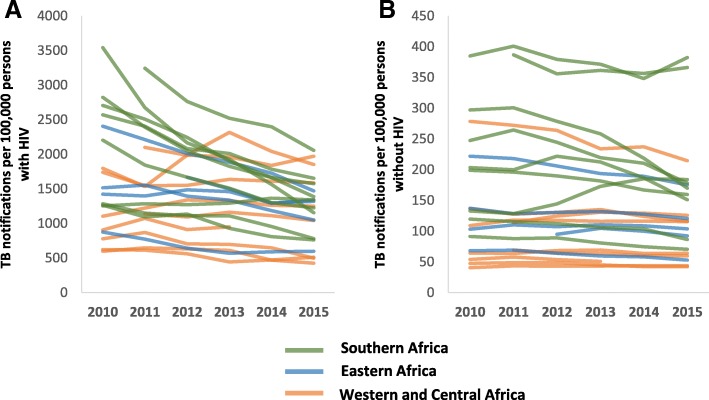
**a** and **b** Trends in estimated TB notification rates among (**a**) PLHIV and (**b**) people without HIV by region in Africa — 23 countries, sub-Saharan Africa, 2010–2015

Among 19 countries with sufficient data to make HIV-stratified TB case notification estimates for 2010, the median estimated TB case notification rate among PLHIV (rounded to the nearest 10) was 1420 cases per 100,000 population (IQR 910–2410 cases per 100,000 population, Fig. 4a). In 2015, all 23 countries had sufficient data, and the median estimated TB notification rate among PLHIV was 1250 cases per 100,000 population (IQR 780–1580 cases per 100,000 population). By contrast, the median estimated TB case notification rate among people without HIV in 2010 was 130 cases per 100,000 population (IQR 70–220 cases per 100,000 population, Fig. 4b). In 2015, it was 120 cases per 100,000 population (IQR 60–180 cases per 100,000 population).

Among all countries in the analysis, the median annual average change in estimated TB notification rates among PLHIV was -5.7% (IQR -6.9 to -1.7%; range -10.9 to +3.3%) compared to a median change of -2.3% (IQR -4.2 to -0.1%; range -7.4 to +9.3) for people without HIV (Table 1). This difference was statistically significant (*p* = 0.0099). TB notification rates declined more among PLHIV than among people without HIV in 16 (70%) countries; in 1 (4%) country, TB notification rates increased in both populations, but the increase was smaller for PLHIV than with people without HIV. In 6 (26%) countries, TB notification rates either decreased less or increased more than TB notification rates in people without HIV; 5 (83%) out of these 6 countries were located in western Africa.

Among 11 countries whose average ART coverage during the analytic period was greater than the median of 36%, the median annual average change in TB notification rates among PLHIV was -6.8% (IQR -7.6 to -5.7%) compared to a median change of -2.1% (IQR -6.0 to 0.7%) for PLHIV in 12 countries whose average ART coverage was less than or equal to the median. This difference was statistically significant (*p* = 0.0106). By contrast, the median annual average change in TB notification rates among people without HIV in the 11 countries with higher ART coverage was -3.9% (IQR -4.7 to -0.1%) compared to a median change of -1.8% (IQR -3.3 to 0.9) for people without HIV in the 12 countries with lower ART coverage. This difference was not statistically significant (*p* = 0.1693).

## Discussion

Based on estimated HIV-stratified TB case notification rates in 23 sub-Saharan African countries, TB notification rates among PLHIV have declined more than among people without HIV, concurrently with the expansion of ART. Additionally, TB notification rates among PLHIV declined more in countries with higher ART coverage. While this analysis was descriptive and could not explore causality, these results are consistent with a population-level effect of ART on decreasing TB incidence among PLHIV.

To our knowledge, this analysis is the first attempt to broadly examine the relationship between ART coverage and TB notification rates among PLHIV across sub-Saharan Africa. Reports from a few countries in eastern and southern Africa [15–17] had previously shown greater declines in TB notification rates among PLHIV than among people without HIV in the era of ART scale-up. Our results suggest this to be the case in other eastern and southern African countries as well. However, in most of the western African countries in our analysis, TB notification rates among PLHIV decreased less than among people without HIV, or actually increased. One possible contributor to this worrisome trend is the low ART coverage in several western African countries; there may be a threshold of ART coverage that is required before population-level declines in TB are observed. Another possible contributor is the fact that western African countries tend to have concentrated rather than generalized HIV epidemics, which leads to greater uncertainty in their adult HIV prevalence estimates. Furthermore, the marginalization of key populations with HIV in these countries may exacerbate their susceptibility to TB despite the availablity of ART [25]. Thus, without more information about the overlap of TB and HIV epidemiology in these settings, estimates made based only on population-level data are more difficult to interpret. Nonetheless, improving the accessibility and acceptability of HIV testing and ART to key populations in these countries may be critical to decreasing TB among PLHIV.

As countries move toward implementing the “Test and Start” policy [26] to treat all PLHIV with ART regardless of CD4 cell count, monitoring the impact of ART scale-up on TB trends will become increasingly important. The ability to do so depends on being able to stratify TB trends by HIV status. As our study demonstrates, it is possible to make crude estimates based on the types of data that are currently available. However, only half the countries in the region had sufficient data of adequate quality, with insufficient HIV testing coverage among TB patients being the most common reason for excluding countries from our analysis. Prioritizing HIV testing for TB patients is thus not only important to ensure appropriate clinical management, but also for monitoring trends in TB incidence among PLHIV.

Although ART is expected to play a critical role in reducing TB incidence among PLHIV, it is not the only important factor. For example, isoniazid preventive therapy (IPT) has been shown to reduce the development of TB, independent of ART, and is increasingly considered an integral component of routine care for PLHIV [27–29]. As scale-up of IPT also occurs across sub-Saharan Africa, monitoring the population-level impact of both ART and IPT on reducing TB incidence will be needed. Furthermore, focusing interventions only on PLHIV will not be sufficient to halt the incidence of TB among PLHIV. People without HIV tend to be a reservoir for TB transmission to PLHIV [1, 6, 30–32], so investment in general TB elimination strategies such as active case-finding to find and treat all cases are crucial to reducing TB incidence among PLHIV. Finally, to better understand the contribution of different factors on reducing TB incidence among PLHIV, research is needed that goes beyond analyzing population-level indicators. For instance, long-term cohort studies of PLHIV can help quantify the impact of IPT and ART delivered in programmatic settings, while molecular epidemiology studies can provide insight into transmission of TB from people without HIV to PLHIV.

Our study was subject to limitations that affect the conclusions that can be drawn from our results. Inherent in a descriptive analysis is the limitation that causal inferences cannot be made. Therefore, although TB incidence declined more among PLHIV than people without HIV, and although the declines in TB incidence were greater among PLHIV in countries with the highest ART coverage, we cannot claim that ART caused the declines. Other factors such as improved nutrition or housing conditions, as well as improved TB programs or health system improvements that occurred in the process of building stronger programs to deliver ART, may have affected TB incidence. It is also possible that increasing IPT coverage could have contributed to the greater declines in TB notification rates observed among PLHIV compared to people without HIV. However, we were unable to assess the potential impact of IPT, or even describe its scale-up, as notification data for the number of people receiving IPT were completely missing for a third of the countries in this analysis, and missing for two thirds of countries for the early years of the analytic period [19].

Our study was also subject to limitations related to the data that were available for analysis. Because we were limited to publicly available country-level data, adequate data were unavailable for over half of the countries in the WHO African region. As a result, our analysis was relatively complete in its coverage of eastern and southern African countries, but highly incomplete for the countries of western and central Africa. This limitation highlights the need to strengthen data in major public domains if we are to assess the impact of recent policy changes moving forward.

Finally, as with all analyses based on TB case notifications, we were unable to account for potential changes in case detection rates over time, or differences in case detection between PLHIV and people without HIV. While we do not know for sure how case detection rates have changed over time, it is likely that improvements in national reporting systems over time have led to increases in the likelihood of people with TB being notified to WHO; therefore, our analysis could underestimate the declines in case notification rates that have occurred. In addition, TB case detection among PLHIV and people without HIV may differ, but the direction of this difference is unknown. For example, given that HIV is a stigmatized disease and the majority of new HIV cases tend to present with advanced immunosuppression [33], delays in TB diagnosis (or missed TB diagnoses altogether) occur more frequently than for people without HIV. By contrast, PLHIV who are in care are routinely screened for TB, while people without HIV are generally not, so case detection among PLHIV may be higher than among people without HIV in some settings. Thus, the quantitative comparison between the case notification rates we estimate for PLHIV and people without HIV must be interpreted with these limitations in mind.

## Conclusion

This analysis suggests encouraging trends that TB notification rates have declined more among PLHIV than among people without HIV from 2010 to 2015. We believe that the expansion of ART has likely contributed to this decline. To further reduce TB incidence among PLHIV, additional scale-up of ART, as well as active case-finding in the general population will be necessary. To monitor the impact of these activities, it will be important to collect data on each intervention as well as routinely assess TB case notification rates stratified by HIV status. And finally, to better understand the factors contributing to changes in TB epidemiology among PLHIV, long-term cohort studies will be important to help interpret trends in programmatic data.

## Abbreviations

ART: Antiretroviral therapy
HIV: Human immunodeficiency virus
IPT: Isoniazid preventive therapy
PLHIV: People living with HIV
TB: Tuberculosis
UNAIDS: Joint United Nations Programme on HIV/AIDS
UNDP: United Nations Development Programme
WHO: World Health Organization
